# Supplementary material for: Role and mechanism of NCAPD3 in promoting malignant behaviors in gastric cancer
Source: Front Pharmacol. 2024 Apr 22;15:1341039. doi: 10.3389/fphar.2024.1341039 (PMC11070777; doi:10.3389/fphar.2024.1341039)
Supplement: Supplementary file 11 [file DataSheet2.ZIP › GSEA/GO_CC/my_analysis.Gsea.1599461408869/GO_RIBOSOME.html]

Details for gene set GO\_RIBOSOME[GSEA]

|  || Dataset | filtered\_dataset.sample\_info.cls#WT\_versus\_NCAPD3\_MUT |
| Phenotype | sample\_info.cls#WT\_versus\_NCAPD3\_MUT |
| Upregulated in class | WT |
| GeneSet | GO\_RIBOSOME |
| Enrichment Score (ES) | 0.3710602 |
| Normalized Enrichment Score (NES) | 1.4125085 |
| Nominal p-value | 0.1185567 |
| FDR q-value | 0.57317585 |
| FWER p-Value | 0.997 |
Table: GSEA Results Summary

  

Fig 1: Enrichment plot: GO\_RIBOSOME      
 Profile of the Running ES Score & Positions of GeneSet Members on the Rank Ordered List

  

| SYMBOL | TITLE | RANK IN GENE LIST | RANK METRIC SCORE | RUNNING ES | CORE ENRICHMENT || 1 | 6191 | RPS4X | 196 | 0.682 | -0.0333 | Yes |
| 2 | 91574 | C12orf65 | 238 | 0.646 | 0.0388 | Yes |
| 3 | 87178 | PNPT1 | 263 | 0.622 | 0.1194 | Yes |
| 4 | 1660 | DHX9 | 510 | 0.469 | 0.0170 | Yes |
| 5 | 78988 | MRPL57 | 557 | 0.441 | 0.0534 | Yes |
| 6 | 26164 | MTG2 | 585 | 0.433 | 0.1021 | Yes |
| 7 | 6208 | RPS14 | 604 | 0.421 | 0.1553 | Yes |
| 8 | 26747 | NUFIP1 | 666 | 0.394 | 0.1736 | Yes |
| 9 | 6182 | MRPL12 | 729 | 0.359 | 0.1857 | Yes |
| 10 | 440275 | EIF2AK4 | 753 | 0.347 | 0.2236 | Yes |
| 11 | 4869 | NPM1 | 772 | 0.336 | 0.2636 | Yes |
| 12 | 79590 | MRPL24 | 800 | 0.320 | 0.2946 | Yes |
| 13 | 51373 | MRPS17 | 817 | 0.306 | 0.3312 | Yes |
| 14 | 6183 | MRPS12 | 838 | 0.280 | 0.3609 | Yes |
| 15 | 27230 | SERP1 | 892 | -0.306 | 0.3711 | Yes |
Table: GSEA details [plain text format]

  

Fig 2: GO\_RIBOSOME      
 Blue-Pink O' Gram in the Space of the Analyzed GeneSet

  

Fig 3: GO\_RIBOSOME: Random ES distribution      
 Gene set null distribution of ES for **GO\_RIBOSOME**
